# Genome-wide analysis of *acetivibrio cellulolyticus* provides a blueprint of an elaborate cellulosome system

**DOI:** 10.1186/1471-2164-13-210

**Published:** 2012-05-30

**Authors:** Bareket Dassa, Ilya Borovok, Raphael Lamed, Bernard Henrissat, Pedro Coutinho, Christopher L Hemme, Yue Huang, Jizhong Zhou, Edward A Bayer

**Affiliations:** 1 Department of Biological Chemistry, The Weizmann Institute of Science, Rehovot, Israel; 2 Department of Molecular Microbiology and Biotechnology, Tel Aviv University, Ramat Aviv, Tel Aviv, Israel; 3 Architecture et Fonction des Macromolecules Biologiques, CNRS and Universite Aix- Marseilles I & II, Marseilles, France; 4 Department of Botany and Microbiology, and Institute for Environmental Genomics, University of Oklahoma, Norman, OK, USA

**Keywords:** Cellulosomics, *Clostridium thermocellum*, Scaffoldin, Cohesin, Dockerin

## Abstract

**Background:**

Microbial degradation of plant cell walls and its conversion to sugars and other byproducts is a key step in the carbon cycle on Earth. In order to process heterogeneous plant-derived biomass, specialized anaerobic bacteria use an elaborate multi-enzyme cellulosome complex to synergistically deconstruct cellulosic substrates. The cellulosome was first discovered in the cellulolytic thermophile, *Clostridium thermocellum*, and much of our knowledge of this intriguing type of protein composite is based on the cellulosome of this environmentally and biotechnologically important bacterium. The recently sequenced genome of the cellulolytic mesophile, *Acetivibrio cellulolyticus,* allows detailed comparison of the cellulosomes of these two select cellulosome-producing bacteria.

**Results:**

Comprehensive analysis of the *A. cellulolyticus* draft genome sequence revealed a very sophisticated cellulosome system. Compared to *C. thermocellum*, the cellulosomal architecture of *A. cellulolyticus* is much more extensive, whereby the genome encodes for twice the number of cohesin- and dockerin-containing proteins. The *A. cellulolyticus* genome has thus evolved an inflated number of 143 dockerin-containing genes, coding for multimodular proteins with distinctive catalytic and carbohydrate-binding modules that play critical roles in biomass degradation. Additionally, 41 putative cohesin modules distributed in 16 different scaffoldin proteins were identified in the genome, representing a broader diversity and modularity than those of *Clostridium thermocellum*. Although many of the *A. cellulolyticus* scaffoldins appear in unconventional modular combinations, elements of the basic structural scaffoldins are maintained in both species. In addition, both species exhibit similarly elaborate cell-anchoring and cellulosome-related gene- regulatory elements.

**Conclusions:**

This work portrays a particularly intricate, cell-surface cellulosome system in *A. cellulolyticus* and provides a blueprint for examining the specific roles of the various cellulosomal components in the degradation of complex carbohydrate substrates of the plant cell wall by the bacterium.

## Background

Plant cell walls are composed of different types of recalcitrant polysaccharides, notably cellulose, which together with lignin form a rigid, stable composite material. Microbial degradation of these polysaccharides and its conversion to sugars is a key step in the carbon cycle, and its subsequent conversion to ethanol is a vital objective for society [1]. One of the major paradigms for efficient degradation of cellulose is a supramolecular, multi-enzyme complex called the cellulosome, which was demonstrated in various bacteria [2-7]. The cellulosome harbors a multiplicity of carbohydrate-active enzymes, i.e., glycoside hydrolases (GHs), carbohydrate esterases (CEs) and polysaccharide lyases (PLs). These include multiple endoglucanases, cellobiohydrolases, xylanases and other degradative enzymes which work synergistically to attack heterogeneous, insoluble cellulose substrates [8-11]. These enzymes are very similar in their mode of action to those of the free enzyme systems of other bacteria and fungi, except that the cellulosomal enzymes contain a dockerin module in place of a carbohydrate-binding module (CBM), which would target the individual enzymes to the substrate. Scaffoldin (Sca), a major cellulosomal subunit, is responsible for organizing the cellulolytic subunits into the complex. The dockerin-borne enzyme subunits are integrated into the scaffoldin subunit via the tenacious protein-protein interaction with multiple copies of cohesin modules. The scaffoldin subunit also contains a single CBM that attaches the entire enzymatic complex (as well as the parent bacterial cell) to the cellulose substrate, thereby enabling efficient synergistic degradation of the substrate.

*Acetivibrio cellulolyticus* is a mesophilic, anaerobic, gram-positive bacterium, known both for its efficient degradation of crystalline cellulose [12-15] and for its distinct protuberant cell surface ultrastructure [16]. A gene cluster of four cellulosomal scaffoldin proteins (ScaA-ScaD) from *A. cellulolyticus* ATCC 33288 was studied during the past decade [17-19]. The primary scaffoldin, ScaA (previously termed CipV), contains a singular intrinsic family-9 glycoside hydrolase (GH) and mediates direct incorporation of the dockerin-containing enzymes through its seven type-I cohesins. It is bound to the cell surface via its C-terminal X-module/dockerin dyad (XDoc) to at least two additional scaffoldins. Thus, ScaA can either interact directly with the ScaD surface-anchoring scaffoldin or it may bind to the ScaC scaffoldin indirectly through a ScaB adaptor scaffoldin [18,20,21]. ScaC and ScaD serve as anchoring scaffoldins, owing to their C- terminal S-layer homology (SLH) modules, but unlike any other scaffolding yet described, the ScaD protein harbors two different types of cohesin (types I and II), which exhibit two divergent dockerin-binding specificities [19]. Thus, only four scaffoldin proteins of the bacterium have been recognized and analyzed prior to sequencing of its genome [22].

Despite the limited genomic information available at the time, a putative model of the cellulosome architecture was proposed, suggesting alternative modes of interactions among the *A. cellulolyticus* scaffoldin components and mechanisms of attachment to the cell surface. Still, the exact model and stoichiometry of the cellulosome arrangement is currently unknown. Original experiments indicated the presence of additional putative cellulosomal enzyme components [18] and scaffoldins [19] which were probed by the ScaC cohesin but were never fully identified.

The expansion of genome sequencing efforts during the past decade has also provided information regarding several cellulosome-producing bacteria [23-26], and their genome-wide comparison has spawned the field of cellulosomics [5], i.e., a general overview of cellulosome-related constituents of a given bacterium. The recent sequencing of the *A. cellulolyticus* genome [22] has thus enabled identification and analysis of numerous additional cellulosomal components, gene regulatory elements, and cell anchoring modules in the bacterium, as documented in this communication. The interrelationship of the *A. cellulolyticus* cellulosome components was further explored by genome-wide comparison of its cellulosomal architecture and subunits with those of *Clostridium thermocellum*.

## Results and discussion

### Multiplicity of scaffoldins and cohesin-containing proteins

**Figure 1:**
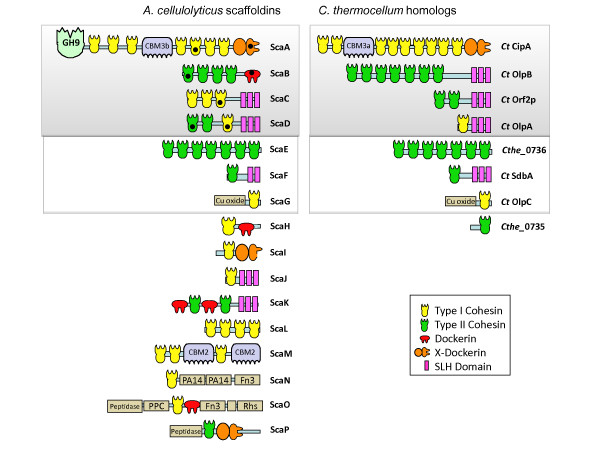
**Modular architecture of the array of scaffoldins identified in the *A. cellulolyticus *CD2 genome and their homologs from *C. thermocellum *ATCC 27405.** Putative *A. cellulolyticus* scaffoldins were identified bioinformatically (see Materials and Methods for their accession numbers). Binding specificities of the indicated (black spots) cohesin and dockerin modules were determined previously [17-19]. The *sca* gene cluster is framed in a shaded box. All proteins have an N-terminal signal peptide except for ScaI. Acronyms: GH9, family-9 glycoside hydrolase; CBM(n), carbohydrate-binding module (family number); Cu, Copper amine oxidase; FN3, Fibronectin type III domain; Peptidase, S8 subtilisin-like peptidase; PPC, bacterial pre-peptidase C-terminal domain; Rhs, Rhs repeat domain. Accession numbers of the *A. cellulolyticus* scaffoldins are: [GenBank: ZP_09464033-30 (ScaA-D), ZP_09465494 (ScaE), ZP_09464236 (ScaF), ZP_09464788 (ScaG), ZP_09462752 (ScaH), ZP_09463446 (ScaI), ZP_09462222 (ScaJ), ZP_09464725 (ScaK), ZP_09464968 (ScaL), ZP_09463433 (ScaM), ZP_09463827 (ScaN), ZP_09462124 (ScaO), ZP_09461865 (ScaP)]. Accession numbers of the *C. thermocellum* scaffoldins are: [GenBank: CAA47840 (CipA), YP_001039467 (OlpB), ABN54275 (Orf2p), YP_001039469 (OlpA), YP_001037164 (Cthe_0736), YP_001037732 (SdbA), YP_001036883 (OlpC) and YP_001037163 (Cthe_0735)]

The *Acetivibrio cellulolyticus* CD2 genome [22] is the largest among the known cellulolytic bacteria (6.1 Mb). Analysis of its recent genome sequence revealed 41 putative cohesin modules, distributed in 16 scaffoldins, some of which have both cohesins and dockerins in the same polypeptide chain (Figure 1 and Additional file 1: Table S1). These include the four genes of the scaffoldin cluster (*scaA, scaB, scaC* and *scaD*), which were originally identified, sequenced and characterized in *A. cellulolyticus* ATCC 33288 [17-19].

The previous publications have indicated that this mesophilic bacterium harbors an intricate cellulosome system, which is characterized by several unique properties that distinguish *A. cellulolyticus* from the archetypical *C. thermocellum* cellulosome: The progression of the ScaA primary scaffoldin, the ScaB adaptor scaffoldin and the ScaC anchoring scaffoldin, with their resident cohesins (7, 4 and 3, respectively), suggests that the resultant fully occupied cellulosome complex would include up to 84 dockerin- containing proteins (enzymes) in addition to the intrinsic ScaA cellulase. The second type of cellulosome complex comprises a divergent anchoring scaffoldin, ScaD, which contains different cohesin specificities: two type-II cohesins that incorporate two ScaA subunits with their complement of dockerin-containing enzymes and a single type-I cohesin that binds a lone dockerin-containing protein.

Comparison of the original *A. cellulolyticus sca* genes which were individually sequenced by conventional methodology [17-19] to those of the newly sequenced genome shows only a few differences (two nucleotide substitutions out of 2601 in the *ScaB* gene [GenBank: ZP_09464032]).

### Modular nature of the cohesin-containing proteins

In the present work, the sequenced *A. cellulolyticus* genome revealed 12 cohesin- containing proteins in addition to the previously known four major scaffoldins encoded by the *sca* gene cluster. Figure 1 presents their modular architecture. All of the proteins listed in the figure, except for ScaI, contain a credible signal peptide, suggesting that these proteins would be secreted.

The cohesin modules exhibit a variety of intriguing sequence features. Like *C. thermocellum*, the 41 cohesins of *A. cellulolyticus* are classified into two types: type I (26 modules) and type II (15 modules). We examined the conservation of the cohesin sequences and compared copies of the various cohesin modules within a given scaffoldin protein, and among the different scaffoldins. The overall sequence similarity among the *A. cellulolyticus* cohesin modules ranges from 41 to 97%. Some scaffoldins contain similar repeats of the same type of cohesin module, whereas others bear a single cohesin. ScaD alone contains a combination of two heterogeneous cohesin types on the same polypeptide chain. As has been experimentally documented [17-19], the cohesin type (i.e., type I or type II cohesin) does not necessarily indicate its binding specificity to a given dockerin. For example, the cohesins from ScaA and ScaC (Figure 1) are all type I according to their sequences, but they bind to different dockerins – the ScaA cohesins bind to the dockerin-bearing enzymes, and the ScaC cohesins bind to the ScaB dockerin.

The combination of S-layer homology (SLH) modules with cohesin modules on the same polypeptide suggests a role for such proteins in anchoring the cellulosome assemblies or specific enzymes to the cell wall of the gram-positive bacterium [27,28].

In addition to the previously described anchoring scaffoldins, ScaC and ScaD, three more proteins which contain SLH modules are now revealed, i.e. ScaF [GenBank: ZP_09464236], ScaJ [ZP_09462222] and ScaK [ZP_09464725]. Of the 37 SLH- containing proteins encoded in the *A. cellulolyticus* genome, ScaK was identified with an SLH module, two dockerins and two cohesin modules. This is the first example of such an architectural arrangement of a cell-surface anchoring scaffoldin that contains both types of cellulosome-related modules.

Uniquely, one cohesin-containing protein also contains two family 2 CBMs, interspacing its type-I cohesins (ScaM, [ZP_09463433]). To our knowledge, this is the first description of a scaffoldin-borne CBM2; all previous CBMs located on scaffoldins have been from family 3. CBM2s have been described as ancillary modules of enzymes and were shown to bind efficiently to cellulose and/or xylan. Thus, their appearance on a scaffoldin may serve to enhance the substrate-binding function of the dockerin- containing enzymes, which bind to this scaffoldin protein via its type-I cohesins. Other cohesins were identified in novel types of scaffoldins which bear FN3 (Fibronectin type III) repeats, PA14 (protective antigen) domain, peptidase or other extracellular modules.

#### Relationship between cohesins of *A. Cellulolyticus* and *C. Thermocellum*

Complex cellulosome architectures were previously proposed for *A. cellulolyticus* and *C. thermocellum,* which are two phylogenetically related Clostridiales species, as implied from their 16 S rRNA analysis [29]. The *C. thermocellum* genome contains 8 cohesin-containing proteins (scaffoldins), whereas *A. cellulolyticus* has twice the number of scaffoldins. The cellulosome system of *C. thermocellum* was selected as the reference strain, since it is the first-identified and best-established multiple-scaffoldin system, which possesses clear similarities to that of *A. cellulolyticus*[4].

Interestingly, three pairs of scaffoldins from both species have the same basic modular organization. Thus, two homologous scaffoldins, *A. cellulolyticus* ScaE [GenBank: ZP_09465494] and *C. thermocellum* Cthe_0736, each consist of seven consecutive type-II cohesins (Figure 1). Likewise, ScaF [GenBank: ZP_09464236] and *C. thermocellum* (Ct) SdbA have a similar architecture comprising a single type-II cohesin followed by an SLH module. Finally, ScaG [GenBank: ZP_09464788] and the cell- surface Ct OlpC [30] both possess a single type-I cohesin, following a unique domain annotated as copper amine oxidase-like [Pfam: PF07833].

**Figure 2:**
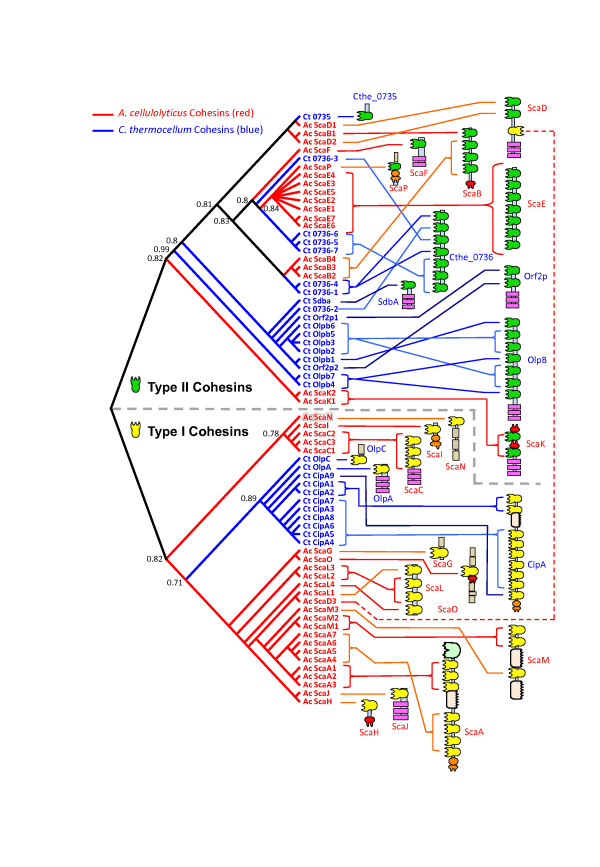
**Relationship of all cohesin modules from *A. cellulolyticus*and *C. thermocellum*.** Sequence-based dendrogram of cohesin modules from *A. cellulolyticus* (red) and *C. thermocellum* (blue). See scheme and key in Figure 1. Only significant bootstrap values are shown

It is important to examine the phylogenetic relationship among the different cohesins within and between the two species, in order to reveal clues regarding their divergence (Figure 2). For example, all seven of the *A. cellulolyticus* ScaE cohesins are similar to each other and are thus clustered together on a single branch of the phylogenetic tree. In contrast, the seven Cthe_0736 cohesins are interwoven on different branches, such that cohesins 1 and 4 are closely related, as are cohesins 5 to 7, indicating domain duplication events in the evolution of this protein. Further diversification of Cthe_0736 is evident in the acquisition of cohesin 2 which bears similarity to divergent type-II cohesins of other *C. thermocellum* anchoring scaffoldins. The seven *A. cellulolyticus* ScaE cohesins appear to be most similar to Cthe_0736 cohesins 3 and 5–7, which presumably suggests a common origin.

The cellulosomes of both species harbor several anchoring proteins, composed of one or more cohesins with SLH modules. For example, ScaF and Ct SdbA have a single type-II cohesin followed by SLH repeats. Yet, their cohesins are clustered on very different branches on the tree (Figure 2), suggesting that their parent proteins are the product of different evolutionary pathways. The ScaF cohesin is closely related to those of ScaE and the above-mentioned Cthe_0736 cohesins, whereas that of Ct SdbA is more similar to those of the other *C. thermocellum* anchoring scaffoldins. In a similar manner, each of the anchoring scaffoldins, ScaJ and Ct OlpA, harbors a single type-I cohesin, located on divergent branches of the phylogenetic tree. As opposed to the type-II cohesins, the relationship among type-I cohesins is more straightforward, where cohesins from each species are clustered on separate branches of the tree.

### Abundance of dockerins in the *A. Cellulolyticus* genome

The *A. cellulolyticus* genome is particularly enriched with dockerin-containing genes, and 143 genes that contain putative dockerin modules were identified. Therefore, *A. cellulolyticus* contains almost twice the number of dockerins as other Clostridial bacteria, such as *Clostridium cellulolyticum* (>60 dockerins) or *Clostridium thermocellum* (>70 dockerins) [23,31,32]. Only the genome of *Ruminococcus flavefaciens* FD-1 is known currently to contain more dockerin-containing genes (>220) [26,33]. Unlike the *R. flavefaciens* dockerins, which are classified into 6 major groups and 11 subgroups [33], the *A. cellulolyticus* dockerins are highly similar, with the exception of six dockerins located downstream of an X module. These latter dockerins have distinctive sequence features compared to the rest of the *A. cellulolyticus* dockerins. Their X-modules are of family X60 [34], which display significant sequence similarity with the X-module at the C-terminus of the *C. thermocellum* CipA scaffoldin. Indeed, several of these X-dockerin pairs are found at the C-terminus of *A. cellulolyticus* scaffoldins (ScaA, ScaP and ScaI). Interestingly, ScaI protein contains an X-dockerin modular dyad with a truncated type-II dockerin at its C-terminus.

**Figure 3:**
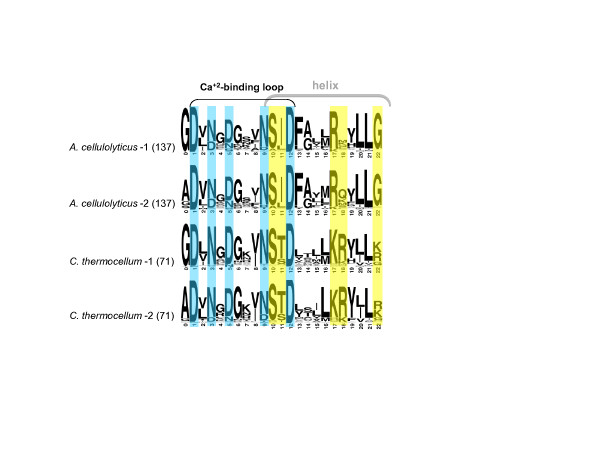
**Sequence conservation pattern of dockerin modules.** The two internal dockerin repeats of *A. cellulolyticus* (based on 137 sequences) and *C. thermocellum* (71 sequences) are represented by sequence logos. Positions of calcium binding residues are shown in cyan, and putative recognition residues are shown in yellow

The characteristic sequence conservation profile [35-37] of the *A. cellulolyticus* dockerin module is shown in Figure 3. The sequence similarity among *A. cellulolyticus* dockerin modules is 53% on average (73% for the most similar dockerins pairs, with no two identical dockerins). Like the dockerins in *C. thermocellum* and unlike those of *R. flavefaciens*, each *A. cellulolyticus* dockerin module contains two canonical Ca + 2 binding repeats, followed by putative helices and linkers. Examination of the putative “recognition” residues of the dockerins, which may participate in their tight binding interface with cohesins, shows a conserved pattern of the two repeated segments wherein S(I/L) residues occupy positions 10 and 11, R(X) positions 17 and 18, and a highly conserved G in position 22 (Figure 3, in yellow). The corresponding positions in the *C.thermocellum* dockerins are S(T/S), K(R/K) and K/R/G, respectively. Position 18 is much less conserved in the *A. cellulolyticus* dockerins than those of *C. thermocellum*, whereas the reverse is true for position 22. Some modifications are evident in position 11 of the *A. cellulolyticus* dockerin sequences. For example, the ScaK scaffoldin contains an N- terminal dockerin with an Asn residue in position 11 of its first dockerin repeat. ScaB dockerin contains Asn residues in both repeats, and instead of the conserved Asn in position 9 it contains a positively charged Lys or Arg residue. In the case of ScaB, these modifications lead to different specificity characteristics, as the dockerin binds selectively to the cohesins of ScaC [18].

### Diversity of dockerin-containing enzymes

*A. cellulolyticus* grows on amorphous and crystalline forms of cellulose, xylans, and cellobiose [38,39]; the bacterium can also be adapted to grow on glucose and xylose [13,40]. Consequently, it was presumed in these early works that the bacterium produces endoglucanases, exoglucanases, β-glucosidases and xylanase activities. Indeed, the present study reveals an intricate array of cellulolytic and hemicellulolytic enzymes in the *A. cellulolyticus* genome, capable of hydrolyzing diverse cellulosic substrates to reducing sugars.

The sequence features of the dockerin-containing enzymes of *A. cellulolyticus* were assessed using the following approach: (i) Like the cohesin-bearing proteins, the dockerin-containing proteins are multimodular in nature, composed of more than one type of module (catalytic, structural, etc.) and sometimes more than one repeat of the same module. The different modular types were therefore enumerated, in order to determine their general distribution among the *A. cellulolyticus* proteins. (ii) Where appropriate, we distinguished between cellulosomal (i.e., those that harbor a dockerin) and non-cellulosomal (without a dockerin) proteins. (iii) We compared the *A. cellulolyticus* proteins with those of *C. thermocellum.*

Among the 143 dockerin-containing proteins, about half (63 proteins) contain one or more known carbohydrate-active CAZyme module(s) [41], and their composition is presented in Table 1 and Additional file 1: Table S1. Because of the multimodular nature of the proteins, some of them contain more than one type of catalytic module, therefore the total sum of catalytic modules in the 63 enzymes is 80 in Table 1 (62 GH-, 13 CE- and/or 5 PL- containing enzymes). Of the 92 GHs, about two-thirds are equipped with dockerins, suggesting that they are recruited to the cellulosome and may thus play a critical role in biomass degradation. Interestingly, the percentage of dockerin-containing GHs in the *A. cellulolyticus* genome is almost identical to that of *C. thermocellum*. The 62 dockerin-containing GHs belong to 19 different families according to the CAZy database (Table 1). As in all known cellulosomes produced by other species, the *A. cellulolyticus* cellulosome contains a single distinctive GH48 enzyme. As in *C. thermocellum,* the *A. cellulolyticus* genome also codes for a second, non-cellulosomal GH48-containing cellulase, as opposed to other characterized cellulosome-producing species that possess only one cellulosomal enzyme. The most abundant GH family is represented by the GH9 enzymes, again like in the *C. thermocellum* cellulosome. This is followed by the GH5 enzymes which are also numerous in both cellulosome-producing species. Of the 21 GH9 enzymes, 10 exhibit a GH9-CBM3 motif that would potentially modulate the activity as in *C. thermocellum* and other cellulolytic bacteria [42-45]. In addition, there are three enzymes that show an extended GH9-CBM3-CBM3 motif, compared to two such enzymes in *C. thermocellum*[46].

**Table 1:** **Comparative distribution of dockerin-containing CAZyme modules in*A. cellulolyticus*vs.*C. thermocellum***

**A. Glycoside Hydrolases families**		**1**	**2**	**3**	**5**	**8**	**9**	**10**	**11**	**13**	**15**	**16**	**18**	**19**	**23**	**26**	**30**	**39**	**43**	**44**	**48**	**51**	**53**	**59**	**74**	**77**	**81**	**94**	**105**	**116**	**124**	**Total**
*A. cellulolyticus*	Genome-wide	2	1	3	16	4	21	4	1	3	1	1	5	2	2	5	3	—	4	1	2	—	1	1	1	1	1	3	1	1	1	92
	Dockerin-containing proteins	—	1	—	12	3	19	4	1	—	—	1	—	—	—	4	4	—	4	1	1	—	1	1	1	—	1	—	1	1	1	62
*C. thermocellum*	Genome-wide	2	1	2	10	1	16	6	1	2	1	2	4	—	2	3	2	1	6	1	2	1	1	—	1	—	1	3	—	—	1	73
	Dockerin-containing proteins	—	1	—	8	1	15	3	1	—	—	1	1	—	—	3	2	1	5	1	1	—	1	—	1	—	1	—	—	—	1	48
**B. Polysaccharide Lyases families**		**1**	**9**	**11**																												
*A. cellulolyticus*	Genome-wide	1	1	3																												5
	Dockerin-containing proteins	1	1	3																												5
*C. thermocellum*	Genome-wide	2	1	1																												4
	Dockerin-containing proteins	2	1	1																												4
**C. Carbohydrate Esterases families**		**1**	**2**	**3**	**4**	**6**	**7**	**8**	**9**	**12**	**14**																					
*A. cellulolyticus*	Genome-wide	2	1	3	4	1	—	1	1	7	—																					20
	Dockerin-containing proteins	2	1	3	1	1	—	1	—	4 (6)	—																					13
*C. thermocellum*	Genome-wide	3	1	2	3	—	1	1	1	2	1																					15
	Dockerin-containing proteins	3	1	1 (2)	1	—	—	1	—	1 (2)	—																					8

Numbers represent proteins which contain one or more modules of the different protein families (glycoside hydrolases, polysaccharide lyases and carbohydrate esterases) as were identified by CAZy. The number of proteins are compared between cellulosomal and non-cellulosomal (genome-wide) proteins. Data are provided for both species. Numbers of modules which appear more than once in the same protein are shown parenthetically.

In one third of the dockerin-containing proteins (46 proteins) we identified modules which are predicted to be associated with extra-cellular proteins (i.e., FN3 modules, Leu-rich repeats, RhsA and PKD domains, see Table 2). Some of these modules are conserved in sequence, but their function is still unknown; some may represent a yet undiscovered enzyme. In this regard, a *C. thermocellum* dockerin-containing protein of previously unknown function was recently demonstrated to be a cellulase [47]. The dockerin-containing proteins of *A. cellulolyticus* are more enriched with such structural and unknown modules than those of *C. thermocellum* (Table 2)*.*

**Table 2:** Summary of protein modules in cellulosomal proteins

	**Cohesins**	**Dockerin- containing proteins**^**a**^	**Modules in dockerin-containing proteins**				
			**Catalytic modules**^**b**^	**Structural modules**^**c**^	**CBMs**^**b**^	**Cohesins**	**Others**^**d**^
*A. cellulolyticus*	41	143 (5)	74	46	53	13	36
*C. thermocellum*	29	73 (3)	51	25	47	9	3

^a^ Numbers in parenthesis indicate the number of X60-dockerin modular pairs in the given species.

^b^ Catalytic modules, such as: GH, PL, CE, and CBMs according to CAZy (http://www.cazy.org/).

^c^ Structural domains are defined in Pfam (http://pfam.sanger.ac.uk/), such as: FN3, GDSL, SNGH, CotH, TRX-like, Kelch-like, SLH, RshA, LRR, PKD.

^d^ Others include Pfam domains such as peptidases, serpins, DUF303, DUF1565, DUF3237.

Many of the GH or CE catalytic modules in the multi-modular proteins are associated with CBMs. In the case of a non-cellulosomal protein, a CBM may serve to deliver the parent catalytic module to a preferred site on the polysaccharide substrate.

Otherwise, an appended CBM may serve to modulate directly the hydrolytic properties of the catalytic module. Table 3 shows the number and distribution of such proteins in the genomes of both bacteria, *A. cellulolyticus* and *C. thermocellum*. Interestingly, 38 of the dockerin-containing enzymes in *A. cellulolyticus* consist of both a catalytic module and a CBM, most of the latter mostly families 3 and 6 (Table 3). In addition, another 12 non- cellulosomal enzymes contain an appended CBM. Although *A. cellulolyticus* contains approximately double the number of dockerin-containing proteins as *C. thermocellum*, the two species have the same number of CBM-appended enzymes (Table 2), and their distribution into different CAZy families largely overlaps.

**Table 3:** **Genome-wide co-occurrence of CBMs together with either GH or CE modules in*A. cellulolyticus*vs.*C. thermocellum***

**CBM families**	**3**	**4**	**6**	**9**	**11**	**13**	**22**	**23**	**27**	**30**	**32**	**34**	**35**	**42**	**44**	**48**	**50**	**54**	**62**
**GH 5**	3/1		2/0		0/1	1/0					1/1								1/0
**GH 9**	19/10	0/2								0/1					1/0				
**GH 10**			2/1	2/2			4/5												
**GH 11**			1/1																
**GH 13**												0/1				2/1			
**GH 16**		0/4																0/1	
**GH 18**	1/0																2/2		
**GH 26**					0/1			1/0	1/0				3/2						
**GH 30**			1/1											0/1					
**GH 39**													0/2						
**GH 43**			2/3			1/1								0/3					
**GH 44**										0/1					1/1				
**GH 48**	1/1																		
**CE 1**			2/1																
**CE 4**			1/1																
**CE 6**			1/0			1/0													1/0
**CE 12**													4/2						

The number of proteins with the combination of the specified modules is noted in the genomes of *A. cellulolyticus* (left) vs. *C. thermocellum* (right).

Even more intriguing are the 10 multi-functional enzymes of *A. cellulolyticus*, which harbor a combination of at least two catalytic modules, including one or two GHs, CEs, PLs and/or glycosyl transferases (GTs), on the same polypeptide (Table 4). In *A. cellulolyticus*, some of these enzymes do not contain a dockerin module. In contrast, *C. thermocellum* codes for 8 multi-functional dockerin-containing enzymes, and *Ruminococcus flavefaciens* FD-1 codes for 18 dockerin-containing multi-functional enzymes. As stated in an earlier section, both genomes encode for two GH48 enzymes – one cellulosomal and another non-cellulosomal. In *C. thermocellum*, there are two separate non-cellulosomal enzymes – Cel48Y (GH48-CBM3b) and the other Cel9I (GH9-CBM3c-CBM3b), whereas in *A. cellulolyticus* the two catalytic modules are fused together into a single polypeptide chain that share a single cellulose-binding CBM3b, thus forming a multi-functional non-cellulosomal enzyme (GH48-GH9-CBM3c-CBM3b, [GenBank:ZP_09464448]).

**Table 4:** **Multifunctional proteins in*A. cellulolyticus*vs.*C. thermocellum***

***A. cellulolyticus***		***C. thermocellum***	
**A. Homologous cellulosomal enzymes**			
**GH11-**CBM6**-**Doc**-CE4**	ZP_09464944	**GH11-**CBM6**-**Doc**-CE4**	Cthe_2972; XynA/U
**PL1-**Doc**-PL9**	ZP_09465691	**PL1**-Doc-CBM35-**PL9**	Cthe_2179
**CE12-**Doc**-CBM35-CE12**	ZP_09463564	**CE12-**Doc**-CBM35-CE12**	Cthe_3141
**CE12-**Doc**-CBM35-CE12**	ZP_09465667		
**B. Non-homologous cellulosomal enzymes**			
**GH5**-CBM6-CBM13-CBM62-Doc-**CE6**	ZP_09463297	CBM30-**GH9**-**GH44**-Doc-CBM44	Cthe_0624; CelJ
**GH5**-Doc-**CE2**	ZP_09464730	CBM22-**GH10**-CBM22-Doc-**CE1**	Cthe_0912; XynY
**CE1**-CBM6-Doc-**GH10**	ZP_09465552	**GH26**-**GH5**-CBM11-Doc	Cthe_1472; CelH
		**GH30**-CBM42-**GH43**-Doc	Cthe_2139
		**CE3**-**CE3**-Doc	Cthe_0798
**C. Non-cellulosomal enzymes**			
**GH48**-**GH9**-CBM3c-CBM3b	ZP_09464448		
**GH18**-**CE4**-GT2	ZP_09465738		
GT84-**GH94**	ZP_09462312		

Domain architectures and their corresponding accession numbers or names are listed above. Catalytic modules are marked in bold. GH, glycoside hydrolase; PL, polysaccharide lyase; CE, carbohydrate esterase; CBM, carbohydrate-binding module; GT, glycosyl transferase; Doc, dockerin; numbers indicate family of the indicated module.

#### Putative cellulosome-related regulatory elements

It is clear that such an elaborate cellulosome system in *A. cellulolyticus* would require a regulatory mechanism by which the bacterium controls expression of its cellulosomal genes. One possible regulator may be inherent in the two types of cohesin modules (i.e., type I and type II), which, like in *C. thermocellum,* signifies at least two divergent specificities of cohesin-dockerin interaction in this species.

Recently, a distinctive system of cellulosome gene regulation was proposed. A carbohydrate-sensing mechanism was described in *C. thermocellum*[48-50], suggesting that a set of putative σ and anti-σ factors are activated by extracellular polysaccharides. Thus, the different components of the cellulosic biomass would be detected extracellularly by corresponding RsgI-borne binding elements (CBMs, GHs, etc.), and appropriate signals are transmitted intracellularly. This in turn was proposed to disassociate the interaction between the intracellular portions of the RsgI-like proteins and complementary σ^I^-like factors, resulting in the release of the σ^I^s, followed by their association with RNA polymerase and transcription of corresponding genes involved in cellulose utilization. Interestingly, analysis of the other known cellulosome-producing bacterial genomes (e.g., *C. cellulolyticum* and *C. cellulovorans*) revealed only a single RsgI-like protein, which lacks a recognizable C-terminal binding element. It therefore appeared that an extensive RsgI-mediated carbohydrate-sensing mechanism is restricted to *C. thermocellum.*

It was thus of interest to evaluate the status of the RsgI-like proteins in *A. cellulolyticus.* Indeed, analysis of the genome revealed multiple copies of genes coding for σ^I^-like factors and their cognate membrane-associated RsgI-like (anti-σ^I^) factors, which may be involved in regulatory mechanisms of cellulosomal and related cellulase genes. Twelve putative σ^I^/RsgI-like proteins were detected in the *A. cellulolyticus* genome (Table 5), as opposed to the eight in *C. thermocellum*. The *A. cellulolyticus* RsgI- like proteins contain predicted C-terminal modules such as CBM3, CBM42, CBM35, PA14-like, but none appeared to contain a GH module like the ones detected in *C. thermocellum*[50]. Significantly, most of the putative σ^I^-like proteins of *A. cellulolyticus* have orthologs in *C. thermocellum*, some of which have been validated experimentally.

**Table 5:** **Putative** σ^**I**^**and anti-**σ^**I**^**regulatory factors in*Acetivibrio cellulolyticus*CD2**

***sigI*-like gene**	***rsgI*-like pair**	**C-terminal sensing domain**	**Ortholog in *C. thermocellum***
ZP_09464729	ZP_09464728	CBM3	Cthe_0403
ZP_09464331	ZP_09464330	CBM3	Cthe_0058
ZP_09466014	ZP_09466013	CBM3	Cthe_0268
ZP_09463653	ZP_09463652	CBM3	Cthe_0058
ZP_09461804	ZP_09461805	CBM42	Cthe_1272
ZP_09463236	ZP_09463235	PA14, CBM35	Cthe_0315
ZP_09464238	ZP_09464237	PA14, PA14	Cthe_1272
ZP_09464575	ZP_09464574	unknown	Cthe_0403
ZP_09464240	ZP_09464239	S1/S6 peptidase	Cthe_0058
ZP_09463889	ZP_09463888	unknown	Cthe_2521
ZP_09466630	ZP_09466631	unknown	Cthe_2974
ZP_09465751	ZP_09465752	unknown	Cthe_2974

For example, the ability of σ^I1^ of *C. thermocellum* to activate the promoters of *sigI1* and a family 48 cellulase, *celS*, was demonstrated in vitro [49]. In addition, the CBMs were shown to bind selectively to typical plant cell wall polysaccharides [48]. Interestingly, genes encoding the σ^I^/RsgI regulatory systems are often found in genomic loci, where they are associated with other genes encoding dockerin- and cohesin-containing proteins (e.g., *celEcel124cel8AscaF* etc.).

The multiple regulatory factors which we identified in *A. cellulolyticus* thus mirror the extensive regulatory system described previously in *C. thermocellum*, and may control the expression levels of cellulosomal and non-cellulosomal genes to reflect changes in the plant cell-wall substrates during the process of decomposition. Moreover, some of these factors may govern processes in the bacterium, which are not directly involved in plant cell wall degradation.

## Conclusions

Early electron microscopy observations of *A. cellulolyticus* demonstrated its particularly elaborate cell surface ultrastructure and its cellulose-degrading activities [16,51]. The availability of its genome sequence has enabled a better appreciation of the complex and modular nature of its cellulosome. Compared to *C. thermocellum*, the cellulosomal architecture of *A. cellulolyticus* is more extensive, encoding twice the number of cohesin- and dockerin-containing proteins, with previously undescribed combinations of protein modules. Yet, certain elements of the basic structural scaffoldins, which dictate the assembly of the various functional carbohydrate-degrading enzymes, are maintained in both species. In addition, both species exhibit elaborate cell-anchoring and gene-regulation systems. Interestingly, the *multiplicity* of σ^I^/RsgI-like proteins may be characteristic of cellulosome-producing bacteria that contain multiple- scaffoldin gene clusters, like *A. cellulolyticus* and *C. thermocellum*, as opposed to those like *C. cellulolyticum,* that contain enzyme-linked gene clusters.

This work provides a blueprint for understanding the cellulosome system of this intriguing cellulose-degrading bacterium and paves the way for studying the specific role of its cellulosomal protein components in the degradation of plant cell-wall carbohydrates. It is clear that the bacterium utilizes a sophisticated system for efficient hydrolysis of crystalline cellulose of the plant cell wall. The cohesin-containing proteins of *A. cellulolyticus* present a broader diversity and modularity than those of *C. thermocellum*, where cohesins are associated in unconventional modular combinations, and their functional roles are yet to be defined.

## Methods

### Genomes source

Draft genome sequences of *Acetivibrio cellulolyticus* CD2 (DSM 1870, ATCC 33288) (30 Dec. 2011), and *Clostridium thermocellum* ATCC 27405 (16 Feb. 2007) were obtained from GenBank (accession: AEDB00000000 and CP000568, respectively). Assembly of *A. cellulolyticus* genome was approached by a combination of sequencing methods, using Sanger, 454-Titanium, 454 Titanium Paired-end and Solexa Paired-end technologies, as detailed in Hemme et al. [22]. The genome assembled into 112 contigs with an average coverage depth of x71.9 +/− 6.3 (interval of depths 9 – 111). Protocols of the *A. cellulolyticus* sequencing methods, assemblies and annotation are detailed in Land et al. [52].

### Sequence identification of cohesins and dockerins

BLAST [53] searches were applied on *A. cellulolyticus* DNA contigs and predicted proteins, using sequences of known cohesin and dockerin modules as queries. All hits above E-value of 10–4 were retrieved and inspected individually, by examining their characteristic sequence features. Obvious dockerin modules were expected to contain two Ca + 2-binding repeats, putative helices and linker regions. Low-scoring hits of dockerins and cohesins were examined by comparing them against known dockerin or cohesin sequences, respectively. Sequence logos of dockerins were created with Weblogo v.2.8.2 (http://weblogo.berkeley.edu/) [54]. Multiple sequence alignment was obtained using CLASTALW [55], with manual corrections when needed.

The scaffoldin genes from *A. cellulolyticus* ATCC 33288 which were manually sequenced [17-19] are ScaA, [GenBank: AF155197]; ScaB, [GenBank: AY221112]; ScaC, [GenBank: AY221113], ScaD, [GenBank: AY221114]). The cohesin dendrogram was generated using PhyML algorithms (with LG substitution model, and default parameters of the Approximate Likelihood-Ratio test) [56] and visualized using TreeView [57].

### Annotation of dockerin-containing enzymes

Dockerin-containing proteins of *A. cellulolyticus* CD2 and *C. thermocellum* ATCC 27405 were annotated by CAZy database (http://www.cazy.org) [41], in order to bioinformatically analyze their catalytic modules. This includes identification of the catalytic modules and their classification into family types, according to sequence conservation, for glycoside hydrolases, carbohydrate esterases, polysaccharide lyases, carbohydrate-binding modules and glycosyl transferases. Additional conserved domains of the proteins were analyzed using the CD-search website (http://www.ncbi.nlm.nih.gov/Structure/cdd/wrpsb.cgi) and the Pfam database (http://pfam.sanger.ac.uk/). Putative cellulosome-related regulatory elements were identified by BLAST searches and sequence similarity using known elements from *C. thermocellum* as queries [48-50].

## Abbreviations

CAZy: Carbohydrate-active enzymes; GH: Glycoside hydrolase; CE: Carbohydrate esterase; PL: Polysaccharide lyase; CBM: Carbohydrate-binding module; GT: Glycosyl transferases; CBM: Carbohydrate-binding module; SLH: S-layer homology; X-doc: X-dockerin; Sca: Scaffoldin.

## Competing interests

The authors declare that they have no competing interests.

## Authors' contributions

BD and EAB conceived of the project and wrote the manuscript. BD, IB, BH and PC analyzed the genome data. CLH, YH and JZ sequenced the genome. BD, RL and EAB wrote the paper. All authors read and approved the final manuscript.

## Supplementary Material

Additional file 1 **Table S1.** Cellulosomal and non-cellulosomal CAZyme proteins in *A. cellulolyticus.* The modular architecture of the indicated proteins show *only* the CAZy-related modules: GH, glycoside hydrolase; PL, polysaccharide lyase; CE, carbohydrate esterase; CBM, carbohydrate-binding module; Doc, dockerin; Coh, cohesin, SLH, S-layer homology modules. Numbers indicate family of the indicated module. **A.** Cohesin-containing proteins. **B.** Dockerin-containing proteins. **C.** Non-cellulosomal CAZymes Click here for file
