# Supplementary material for: Genome-wide analysis of acetivibrio cellulolyticus provides a blueprint of an elaborate cellulosome system
Source: BMC Genomics. 2012 May 30;13:210. doi: 10.1186/1471-2164-13-210 (PMC3413522; doi:10.1186/1471-2164-13-210)
Supplement: Additional file 1 — Table S1. Cellulosomal and non-cellulosomal CAZyme proteins in A. cellulolyticus. The modular architecture of the indicated proteins show only the CAZy-related modules: GH, glycoside hydrolase; PL, polysaccharide lyase; CE, carbohydrate esterase; CBM, carbohydrate-binding module; Doc, dockerin; Coh, cohesin, SLH, S-layer homology modules. Numbers indicate family of the indicated module. A. Cohesin-containing proteins. B. Dockerin-containing proteins. C. Non-cellulosomal CAZymes [file 1471-2164-13-210-S1.doc]

Supplementary Table S1. **Cellulosomal and non-cellulosomal CAZyme proteins in *A. cellulolyticus.*** The modular architecture of the indicated proteins show ***only*** the CAZy-related modules: GH, glycoside hydrolase; PL, polysaccharide lyase; CE, carbohydrate esterase; CBM, carbohydrate-binding module; Doc, dockerin; Coh, cohesin, SLH, S-layer homology modules. Numbers indicate family of the indicated module.

**A. Cohesin-containing proteins**

|  | **Cohesin**  **name** | | **CAZy modules** | | **Accession number** | |
| --- | --- | --- | --- | --- | --- | --- |
|  | ScaA | | GH9-COH1-COH1-COH1-CBM3b-COH1-COH1-  COH1-COH1-DOC | | ZP_09464033 | |
|  | ScaB | | COH2-COH2-COH2-COH2-DOC | | ZP_09464032 | |
|  | ScaC | | COH1-COH1-COH1-SLH-SLH-SLH | | ZP_09464031 | |
|  | ScaD | | COH2-COH2-COH1-SLH-SLH-SLH | | ZP_09464030 | |
|  | ScaE | | COH2-COH2-COH2- COH2-COH2-COH2-COH2 | | ZP_09465494 | |
|  | ScaF | | COH2-SLH-SLH | | ZP_09464236 | |
|  | ScaG | | COH1 | | ZP_09464788 | |
|  | ScaH | | COH1-DOC | | ZP_09462752 | |
|  | ScaI | | COH1-DOC | | ZP_09463446 | |
|  | ScaJ | | COH1-SLH-SLH-SLH | | ZP_09462222 | |
|  | ScaK | | DOC-COH2-DOC-COH2-SLH-SLH-SLH | | ZP_09464725 | |
|  | ScaL | | COH1-COH1-COH1-COH1 | | ZP_09464968 | |
| ScaM | | COH1-COH1-CBM2-COH-CBM2 | | ZP_09463433 | |  |
| ScaN | | COH1 | | ZP_09463827 | |  |
| ScaO | | COH1-DOC | | ZP_09462124 | |  |
| ScaP | | COH2-DOC | | ZP_09461865 | |  |

**B. Dockerin-containing proteins**

|  | | **CAZy modules** | | **Accession number** | | |  |
| --- | --- | --- | --- | --- | --- | --- | --- |
|  | | GH10-CBM6-DOC | | ZP_09465528 | | |  |
|  | | GH116-DOC | | ZP_09462323 | | |  |
|  | | GH11-CBM6-DOC-CE4 | | ZP_09464944 | | |  |
|  | | GH16-DOC | | ZP_09463643 | | |  |
|  | | GH26-DOC | | ZP_09462417 | | |  |
|  | | GH2-DOC | | ZP_09462051 | | |  |
|  | | GH30-CBM6-DOC | | ZP_09464584 | | |  |
|  | | GH30-DOC | | ZP_09463662 | | |  |
|  | | GH30-DOC | | ZP_09462052 | | |  |
|  | | GH43-CBM13-DOC | | ZP_09464251 | | |  |
|  | | GH43-CBM6-DOC | | ZP_09461799 | | |  |
|  | GH43-CBM6-DOC | | ZP_09465529 | | |  | |
|  | GH43-DOC | | ZP_09461795 | | |  | |
|  | GH44-DOC-CBM44 | | ZP_09462907 | | |  | |
|  | GH48-DOC | | ZP_09463651 | | |  | |
|  | GH53-DOC | | ZP_09463661 | | |  | |
|  | GH59-CBM6-DOC | | ZP_09461802 | | |  | |
|  | GH5-CBM32-DOC | | ZP_09466634 | | |  | |
|  | GH5-CBM6-CBM13-CBM62-DOC-CE6 | | ZP_09463297 | | |  | |
|  | GH5-DOC | | ZP_09466195 | | |  | |
|  | GH5-DOC | | ZP_09462316 | | |  | |
|  | GH5-DOC | | ZP_09464781 | | |  | |
|  | GH5-DOC | | ZP_09463540 | | |  | |
|  | GH5-DOC | | ZP_09466370 | | |  | |
|  | GH5-DOC | | ZP_09466096 | | |  | |
|  | GH5-DOC | | ZP_09465414 | | |  | |
|  | GH5-DOC | | ZP_09462401 | | |  | |
|  | GH5-DOC-CE2 | | ZP_09464730 | | |  | |
|  | GH74-DOC | | ZP_09463654 | | |  | |
|  | GH81-DOC | | ZP_09462356 | | |  | |
|  | GH8-DOC | | ZP_09462868 | | |  | |
|  | GH8-DOC | | ZP_09466015 | | |  | |
|  | GH8-DOC | | ZP_09462762 | | |  | |
|  | GH9-CBM3-CBM3-DOC | | ZP_09464491 | | |  | |
|  | GH9-CBM3-CBM3-DOC | | ZP_09465391 | | |  | |
|  | GH9-CBM3-CBM3-DOC | | ZP_09463149 | | |  | |
|  | GH9-CBM3-DOC | | ZP_09464736 | | |  | |
|  | GH9-CBM3-DOC | | ZP_09464751 | | |  | |
|  | GH9-CBM3-DOC | | ZP_09464334 | | |  | |
|  | GH9-CBM3-DOC | | ZP_09466016 | | |  | |
|  | GH9-CBM3-DOC | | ZP_09461783 | | |  | |
|  | GH9-CBM3-DOC | | ZP_09465501 | | |  | |
|  | GH9-CBM3-DOC | | ZP_09465390 | | |  | |
|  | GH9-CBM3-DOC | | ZP_09463915 | | |  | |
|  | GH9-CBM3-DOC | | ZP_09463916 | | |  | |
|  | GH9-CBM3-DOC | | ZP_09464033 | | |  | |
|  | GH9-CBM3-DOC | | ZP_09464070 | | |  | |
|  | GH9-DOC | | ZP_09466026 | | |  | |
|  | GH9-DOC | | ZP_09466025 | | |  | |
|  | GH9-DOC | | ZP_09463205 | | |  | |
|  | GH9-DOC | | ZP_09464794 | | |  | |
|  | GH9-DOC | | ZP_09462798 | | |  | |
| CBM11-DOC | | | | | ZP_09463286 | | |
| CBM13-DOC | | | | | ZP_09461798 | | |
| CBM22-CBM22-CBM22-GH10-CBM9-  CBM9-DOC | | | | | ZP_09461840 | | |
| CBM22-GH10-DOC | | | | | ZP_09464359 | | |
| CBM27-GH26-CBM23-DOC | | | | | ZP_09465765 | | |
| CBM35-GH26-DOC | | | | | ZP_09465727 | | |
| CBM35-GH26-DOC | | | | | ZP_09464200 | | |
| CBM35-GH26-DOC | | | | | ZP_09465411 | | |
| CBM3-CBM3-DOC | | | | | ZP_09465673 | | |
| CBM3-GH5-DOC | | | | | ZP_09462893 | | |
| CBM4-DOC | | | | | ZP_09464449 | | |
| CBM6-DOC | | | | | ZP_09461801 | | |
| CBM6-DOC | | | | | ZP_09461800 | | |
| CE12-DOC | | | | | ZP_09462063 | | |
| CE12-DOC-CBM35-CE12 | | | | | ZP_09463564 | | |
| CE12-DOC-CBM35-CE12 | | | | | ZP_09465667 | | |
| CE1-CBM6-DOC | | | | | ZP_09465527 | | |
| CE1-CBM6-DOC-GH10 | | | | | ZP_09465552 | | |
| CE3-DOC | | | | | ZP_09465728 | | |
| CE3-DOC | | | | | ZP_09465191 | | |
| CE3-DOC | | | | | ZP_09466148 | | |
| CE8-DOC | | | | | ZP_09464958 | | |
| DOC | | | | | ZP_09464695 | | |
| DOC | | | | | ZP_09464696 | | |
| DOC | | | | | ZP_09464697 | | |
| DOC | | | | | ZP_09464727 | | |
| DOC | | | | | ZP_09462977 | | |
| DOC | | | | | ZP_09462867 | | |
| DOC | | | | | ZP_09462851 | | |
| DOC | | | | | ZP_09462847 | | |
| DOC | | | | | ZP_09465841 | | |
| DOC | | | | | ZP_09466020 | | |
| DOC | | | | | ZP_09466012 | | |
| DOC | | | | | ZP_09466004 | | |
| DOC | | | | | ZP_09461865 | | |
| DOC | | | | | ZP_09461806 | | |
| DOC | | | | | ZP_09461791 | | |
| DOC | | | | | ZP_09461790 | | |
| DOC | | | | | ZP_09465192 | | |
| DOC | | | | | ZP_09465177 | | |
| DOC | | | | | ZP_09466412 | | |
| DOC | | | | | ZP_09462507 | | |
| DOC | | | | | ZP_09463232 | | |
| DOC | | | | | ZP_09466644 | | |
| DOC | | | | | ZP_09466625 | | |
| DOC | | | | | ZP_09466613 | | |
| DOC | | | | | ZP_09462752 | | |
| DOC | | | | | ZP_09465617 | | |
| DOC | | | | | ZP_09465590 | | |
| DOC | | | | | ZP_09465579 | | |
| DOC | | | | | ZP_09463565 | | |
| DOC | | | | | ZP_09463582 | | |
| DOC | | | | | ZP_09464158 | | |
| DOC | | | | | ZP_09464187 | | |
| DOC | | | | | ZP_09464195 | | |
| DOC | | | | | ZP_09464950 | | |
| DOC | | | | | ZP_09465549 | | |
| DOC | | | | | ZP_09465540 | | |
| DOC | | | | | ZP_09465526 | | |
| DOC | | | | | ZP_09465516 | | |
| DOC | | | | | ZP_09463023 | | |
| DOC | | | | | ZP_09465353 | | |
| DOC | | | | | ZP_09462050 | | |
| DOC | | | | | ZP_09463970 | | |
| DOC | | | | | ZP_09464120 | | |
| DOC | | | | | ZP_09465380 | | |
| DOC | | | | | ZP_09465366 | | |
| DOC | | | | | ZP_09463099 | | |
| DOC | | | | | ZP_09463098 | | |
| DOC | | | | | ZP_09462124 | | |
| DOC | | | | | ZP_09463876 | | |
| DOC | | | | | ZP_09463518 | | |
| DOC | | | | | ZP_09465413 | | |
| DOC | | | | | ZP_09463152 | | |
| DOC | | | | | ZP_09464583 | | |
| DOC | | | | | ZP_09465859 | | |
| DOC-CBM32 | | | | | ZP_09466133 | | |
| DOC-CE12 | | | | | ZP_09462064 | | |
| DOC-DOC | | | | | ZP_09465996 | | |
| DOC-GH105 | | | | | ZP_09463369 | | |
| DOC-GH124 | | | | | ZP_09464332 | | |
| DOC-PL11 | | | | | ZP_09463921 | | |
| DOC-PL11 | | | | | ZP_09464380 | | |
| PL11-DOC | | | | | ZP_09465328 | | |
| PL1-DOC-PL9 | | | | | ZP_09465691 | | |

**C. Non-cellulosomal CAZymes**

|  | **CAZy modules** | **Accession number** | |  |
| --- | --- | --- | --- | --- |
|  | CBM16 | ZP_09464013 | |  |
|  | CBM16 | ZP_09464014 | |  |
|  | CBM16-GT39 | ZP_09461815 | |  |
|  | CBM16-GT39 | ZP_09461815 | |  |
|  | CBM2 | ZP_09464749 | |  |
|  | CBM2 | ZP_09465611 | |  |
|  | CBM25 | ZP_09462366 | |  |
|  | CBM25 | ZP_09465597 | |  |
|  | CBM25 | ZP_09464150 | |  |
|  | CBM25 | ZP_09466342 | |  |
|  | CBM25 | ZP_09464384 | |  |
|  | CBM2-CBM2 | ZP_09463433 | |  |
|  | CBM3 | ZP_09464728 | |  |
|  | CBM3 | ZP_09465796 | |  |
|  | CBM3 | ZP_09464330 | |  |
|  | CBM3 | ZP_09466013 | |  |
|  | CBM3 | ZP_09463652 | |  |
|  | CBM3 | ZP_09466062 | |  |
|  | CBM3 | ZP_09466191 | |  |
|  | CBM3 | ZP_09466151 | |  |
|  | CBM32 | ZP_09463920 | |  |
|  | CBM32 | ZP_09463919 | |  |
|  | CBM32-CBM32 | ZP_09463308 | |  |
|  | CBM32-CBM35-CBM35 | ZP_09465209 | |  |
|  | CBM32-CBM3-CBM3 | ZP_09464930 | |  |
|  | CBM35 | ZP_09463235 | |  |
|  | CBM3-CBM3 | ZP_09466584 | |  |
|  | CBM3-CBM3-CBM3 | ZP_09465760 | |  |
|  | CBM3-GH18 | ZP_09463685 | |  |
|  | CBM3-SLH-SLH-SLH | ZP_09462974 | |  |
|  | CBM42 | ZP_09461805 | |  |
|  | CBM48-GH13 | ZP_09462460 | |  |
| CBM48-GH13 | | | ZP_09463887 | |
| CBM50 | | | ZP_09464669 | |
| CBM50 | | | ZP_09466509 | |
| CBM50 | | | ZP_09463364 | |
| CBM50 | | | ZP_09465470 | |
| CBM50 | | | ZP_09465467 | |
| CBM50 | | | ZP_09464912 | |
| CBM50-CBM50 | | | ZP_09465614 | |
| CBM50-CBM50 | | | ZP_09465296 | |
| CBM50-CBM50 | | | ZP_09463009 | |
| CBM50-CBM50 | | | ZP_09465449 | |
| CBM50-CBM50-GH18 | | | ZP_09463008 | |
| CBM51-CBM51 | | | ZP_09463415 | |
| CBM6-CBM3 | | | ZP_09463270 | |
| CBM6-CBM6-SLH-SLH | | | ZP_09464791 | |
| CBM9 | | | ZP_09465587 | |
| CE12 | | | ZP_09462850 | |
| CE4 | | | ZP_09466499 | |
| CE4 | | | ZP_09462373 | |
| CE9 | | | ZP_09465693 | |
| GH1 | | | ZP_09464739 | |
| GH1 | | | ZP_09466280 | |
| GH13 | | | ZP_09465432 | |
| GH15 | | | ZP_09462944 | |
| GH18 | | | ZP_09463642 | |
| GH18 | | | ZP_09463513 | |
| GH18-CE4-GT2 | | | ZP_09465738 | |
| GH19 | | | ZP_09463701 | |
| GH19 | | | ZP_09463699 | |
| GH23 | | | ZP_09462679 | |
| GH23 | | | ZP_09466542 | |
| GH3 | | | ZP_09462790 | |
| GH3 | | | ZP_09465342 | |
| GH3 | | | ZP_09464435 | |
| GH48-GH9-CBM3-CBM3 | | | ZP_09464448 | |
| GH5 | | | ZP_09465173 | |
| GH5 | | | ZP_09464915 | |
| GH5 | | | ZP_09464067 | |
| GH5-CBM3-CBM3 | | | ZP_09464466 | |
| GH77 | | | ZP_09464780 | |
| GH8 | | | ZP_09462484 | |
| GH9 | | | ZP_09463145 | |
| GH94 | | | ZP_09463104 | |
| GH94 | | | ZP_09463103 | |
| GT39-CBM16 | | | ZP_09465789 | |
| GT84-GH94 | | | ZP_09462312 | |
